# Quantifying Short-Term Dynamics of Parkinson’s Disease Using Self-Reported Symptom Data From an Internet Social Network

**DOI:** 10.2196/jmir.2112

**Published:** 2013-01-24

**Authors:** Max Little, Paul Wicks, Timothy Vaughan, Alex Pentland

**Affiliations:** ^1^Human Dynamics GroupMedia LabMassachusetts Institute of TechnologyCambridge, MAUnited States; ^2^Research and DevelopmentPatientsLikeMe Inc.Cambridge, MAUnited States

**Keywords:** Parkinson’s disease, social networks, medical informatics, symptoms, pharmacodynamics

## Abstract

**Background:**

Parkinson’s disease (PD) is an incurable neurological disease with approximately 0.3% prevalence. The hallmark symptom is gradual movement deterioration. Current scientific consensus about disease progression holds that symptoms will worsen smoothly over time unless treated. Accurate information about symptom dynamics is of critical importance to patients, caregivers, and the scientific community for the design of new treatments, clinical decision making, and individual disease management. Long-term studies characterize the typical time course of the disease as an early linear progression gradually reaching a plateau in later stages. However, symptom dynamics over durations of days to weeks remains unquantified. Currently, there is a scarcity of objective clinical information about symptom dynamics at intervals shorter than 3 months stretching over several years, but Internet-based patient self-report platforms may change this.

**Objective:**

To assess the clinical value of online self-reported PD symptom data recorded by users of the health-focused Internet social research platform PatientsLikeMe (PLM), in which patients quantify their symptoms on a regular basis on a subset of the Unified Parkinson’s Disease Ratings Scale (UPDRS). By analyzing this data, we aim for a scientific window on the nature of symptom dynamics for assessment intervals shorter than 3 months over durations of several years.

**Methods:**

Online self-reported data was validated against the gold standard Parkinson’s Disease Data and Organizing Center (PD-DOC) database, containing clinical symptom data at intervals greater than 3 months. The data were compared visually using quantile-quantile plots, and numerically using the Kolmogorov-Smirnov test. By using a simple piecewise linear trend estimation algorithm, the PLM data was smoothed to separate random fluctuations from continuous symptom dynamics. Subtracting the trends from the original data revealed random fluctuations in symptom severity. The average magnitude of fluctuations versus time since diagnosis was modeled by using a gamma generalized linear model.

**Results:**

Distributions of ages at diagnosis and UPDRS in the PLM and PD-DOC databases were broadly consistent. The PLM patients were systematically younger than the PD-DOC patients and showed increased symptom severity in the PD off state. The average fluctuation in symptoms (UPDRS Parts I and II) was 2.6 points at the time of diagnosis, rising to 5.9 points 16 years after diagnosis. This fluctuation exceeds the estimated minimal and moderate clinically important differences, respectively. Not all patients conformed to the current clinical picture of gradual, smooth changes: many patients had regimes where symptom severity varied in an unpredictable manner, or underwent large rapid changes in an otherwise more stable progression.

**Conclusions:**

This information about short-term PD symptom dynamics contributes new scientific understanding about the disease progression, currently very costly to obtain without self-administered Internet-based reporting. This understanding should have implications for the optimization of clinical trials into new treatments and for the choice of treatment decision timescales.

## Introduction

Parkinson’s disease (PD) is a relatively common, progressive neurological disorder affecting approximately 0.3% of the general population in industrialized countries [1]. It generally affects people over 60 years, but rarely younger people under the age of 40 years also develop the disease. PD is considered a movement disorder (ie, it affects the ability to perform normal voluntary motion), but patients also experience cognitive impairment and emotional/mood disturbances. The classic movement symptoms of PD include exaggerated tremor, rigidity, and slow or hesitant motion. These movement problems often have a substantial negative impact on the ability of the patient to perform essential everyday activities, such as bathing, dressing, turning in bed, walking unaided, and getting up from a sitting position. The cause of PD is currently thought to be the loss of dopaminergic neurons in an area of the brain known as the substantia nigra. PD is incurable, and there are no absolutely conclusive diagnostic tests. The most accurate diagnosis based on behavioral symptoms achieves, at best, 90% accuracy when compared to postmortem pathological examination [2].

The mortality rate of patients with the disease is significantly increased relative to healthy people [1]. There are a few approaches to treating the symptoms of PD. The first line of defense is the drug levodopa that replenishes dopamine in the substantia nigra, which reduces the severity of movement symptoms. However, this drug tends to become less effective over time and can also lead to severe side effects, such as involuntary movements (dyskinesias). Surgical treatments, such as deep brain stimulation, have been shown to be effective for many patients who do not respond or have ceased to respond to drug treatments. Current scientific understanding holds that the severity of PD symptoms will smoothly increase over time, faster at first and often leveling out in the later stages [3].

Trials for new treatments and assessing the effectiveness of treatments require objective data about symptom severity. A coarse quantitative measure of symptom severity is the Hoehn and Yahr (HY) ordinal scale [4] that assigns a number from 0 to 5, with 0 being healthy and 5 denoting severe disability. This has been largely supplanted by the ordinal Unified Parkinson’s Disease Rating Scale (UPDRS) (version 3.0) [5] and associated tests [6], which are more time consuming and expensive to administer, but are more precise. The most commonly used parts of the UPDRS (Parts I, II, and III) range on a scale from 0 (healthy) to 176 (severe disability) [5], although a simple and accurate formula exists to predict HY from UPDRS [7].

The UPDRS values have been collected for patients at all stages of the disease, and there is substantial research data available on PD symptom progression quantified on this scale. This kind of data has been used to calibrate models of PD symptom progression over the course of years to decades [3]. However, the full UPDRS is a complex test that requires expertise to administer (even if that expertise can be taught to general medical personnel [5]), attendance of the patient in the clinic, and the average time for administration of the full test is approximately 17 minutes [8]. Unfortunately, these difficulties mean that it is usually prohibitive to objectively score PD symptom severity on timescales shorter than 3 months (low frequency). Since most longitudinal UPDRS data is low frequency, objective information about symptom dynamics occurring on a shorter timescale than 3 months (high-frequency data) is lacking.

There are many clinical situations in which high-frequency symptom dynamics would be useful. For example, in testing new drug treatments there is a trade-off between minimizing exposure to the novel drug to reduce the risk of unknown side effects and maximizing the opportunity to detect significant changes in symptoms. This temporal trade-off cannot be optimized on a quantitative basis without high-frequency data upon which to base the statistical analysis. Similar issues arise in diagnosis where PD is suspected. If, in conjunction with movement symptoms on 1 side of the body only, taking levodopa leads to a reduction in symptom severity, the patient is highly likely to have PD [2]. However, there is still a non-negligible chance that the patient has some other neurological disorder with PD-like symptoms, such as progressive supranuclear palsy. This disease can progress very rapidly, so it is important to diagnose this quickly. Thus, the window of this “exploratory” prescription of levodopa for differential diagnosis must be made as short as possible. However, it should not be so short that rapid, natural fluctuations in symptoms confound proper diagnosis.

Recently, health-focused Internet websites have been established that allow users to track their disease progression by using surveys and other remote monitoring devices, for example. We obtained the UPDRS data from PD users of the PatientsLikeMe website [9], which has recruited over 6000 PD patients worldwide since 2007. Some of these patients are particularly dedicated diarists who have documented their symptoms on a regular basis over a number of years. The result is an unprecedented, high-frequency symptom dataset that has the potential to be used to address some of the shortcomings of existing low-frequency clinical data. For example, if the data are sufficiently accurate, it could be used to supplement in-clinic checkups between visits. Similar data were used for another neurological disease (amyotrophic lateral sclerosis) to refute the idea that lithium carbonate slowed the progression of that disease [10]. The purpose of this study is an exploratory investigation into the high-frequency dynamics and other properties of this novel PD dataset to assess the clinical value of these data.

## Methods

### Patient Recruitment and Data Collection

The main outcomes of this study were quantified by using the UPDRS. This scale consists of 5 parts: Part I covers cognitive, behavioral, and mood symptoms; Part II evaluates activities of daily living; Part III measures motor symptom severity; and Parts IV and V contain HY stage and an evaluation of daily living activities on the Schwab and England scale [6]. Parts I to III contain separate sections, each with a score ranging from 0 (no symptoms) to 4 (severe symptoms). Part I has 4 sections, and Part II has 13 sections.

Two data sources were used: the PatientsLikeMe (PLM) dataset and the Parkinson’s Disease Data and Organizing Center (PD-DOC) dataset [11]. The PLM data were used to provide long-term quantification of individual symptoms occurring on a timescale shorter than 3 months. The data are entirely self-reported. Users sign up to the website where they can enter demographic details, information about their disease course and symptoms, and their treatment history. Specifically, we collected age, gender, treatment status, HY staging, and UPDRS Parts I (mentation, behavior, and mood) and II (activities of daily living) information. Part III of the UPDRS (motor symptoms) was excluded because the collection of this data was deemed not suitable for self-report. Not all self-reported symptoms were accompanied by treatment status indications.

At the time of preparation of this manuscript, the PLM dataset contained 6074 PD patients, of which 2931 completed at least 1 UPDRS survey and entered their birth date and date of diagnosis. Patients were included in this study if they reported at least 15 UPDRS scores with a maximum average UPDRS reporting interval of 65 days between reports. This led to 100 patients being included in this study, and a mean of 29 (SD 14) symptom self-reports per patient (total of 2896 reports), with a reporting interval mean of 45 days (SD 12). The mean age of the selected patients was 54 years (SD 9) at diagnosis, of which 52 were female, 48 male. Patients began self-reporting symptoms approximately 1 year after diagnosis, on average. The total time interval covered by self-reporting per patient, from the first report to the last, was 3.1 years (SD 0.8), and all reports were prospective (after date of joining the website). Patients contributing to the PLM data agreed to the terms and conditions of the website when they enrolled, which included granting permission to PLM to use their medical data for research purposes [12]. Qualitatively, the PLM dataset represents a large number of frequent Part I and II UPDRS reports and treatment status across a medium-size cohort of young to middle-aged patients.

The PD-DOC dataset contains data on PD patients from multiple clinical centers in the United States across several trials with data collected by clinicians over the period from 2006 to 2011 to aid the process of statistical analysis of PD and for the design and planning of clinical trials into treatments. In this study, it was used as a reference dataset to verify the PLM data and to provide background data on PD. Data collection was coordinated by the University of Rochester, Rochester, NY. The set represents UPDRS symptom reports from 564 individuals with PD, of which 200 were female and 364 male, and a mean age of 59 years (SD 10) at diagnosis. In PD, during the day there will be “on” periods when the symptoms of PD are suppressed, to a greater or lesser degree, by the treatment, and “off” periods when the full symptoms reoccur even while taking treatment. In the “on” state, 1612 UPDRS scores were recorded and 354 were recorded in the “off” state. There were a mean of 2.9 (SD 0.9) symptom reports, covering an average of 1.9 years (SD 0.9) per patient. Ethical approval was obtained from the independent review boards of each US medical center contributing patient details to the dataset. In contrast to the PLM dataset, PD-DOC can be described as data from a large number of middle-aged to older patients with clinical UPDRS reports collected on an infrequent basis.

### Validating the PatientsLikeMe Dataset

At the outset, the concept of symptom self-reporting may raise data reliability questions, primarily because it could be suspected that untrained nonclinical raters may be more prone to certain systematic errors or biases than trained clinical raters. For example, they may tend to be biased toward repeating previous measurements, or may have more inconsistent interpretations of specific questions across tests than trained clinical staff. Previous research has shown that when PD patients without dementia self-report UPDRS Parts I and II scores, the scores are consistent with those assessed by the neurologist assessing them [13]. To our knowledge, there have been no similar assessments into the reliability of self-reported UPDRS Parts I and II scoring conducted online under nonclinical circumstances.

To address this issue, we compared the PLM dataset against the PD-DOC data that we considered to be a gold standard clinical reference set. The distributions of UPDRS Parts I and II values and ages at diagnosis were compared visually on quantile-quantile (q-q) plots: if the distributions were of the same form (ie, the same up to a transformation of location and scale, typically the mean and standard deviation), then on the q-q plot the data will lie, approximately, on a straight line [14]. In addition, if the location and scale parameters are the same, the data will lie on a line with a slope equal to 1. Numerical comparisons were made by using the 2-sample Kolmogorov-Smirnov (K-S) test applied to the *z*-scored data (ie, data in which the mean was removed and then divided by the standard deviation). This high-precision test was applied to quantify the results of the visual q-q plot analysis.

### Trend Estimation

To analyze the dynamics of PD over short time periods, it is necessary to remove the effect of trends that occurred due to the natural progression of the disease over that timescale. One widespread approach to modeling disease progression is the use of hierarchical mixed-effects models [15]. These are commonly applied in pharmacodynamics studies [16]. Considerable effort over the preceding decades has increased the sophistication of these models from their origins in simple linear mixed-effects models by incorporating additional features, such as smooth [15] or abrupt nonlinearities in progression [3], nonparametric progression curves [17], and more recently, clustering of individuals into arbitrary groupings using nonparametric Bayes techniques [18]. In PD, pharmacodynamic studies have fitted the smooth, Gompertz sigmoidal curve as a model for progression over the lifetime of the patient with parameters estimated on low-frequency data [3].

A predominant feature of these models is pooling data between subjects. Drawing on specific knowledge about underlying physiological processes (eg, in virology, the mechanism of viral infection of cell populations can give a biologically plausible functional form for the curve). Then the problem becomes one of estimating the parameters for the curve, also known as a regression problem. When there is insufficient progression data about each individual to get reliable (low variance) individual parameter estimates, a global model that fits the data pooled over all individuals can be more reliable, but biased with respect to each individual. By assuming that the individual regression parameters are random variables, it is possible to form compromise parameter estimates by using an appropriate mix of the global and individual models: this is the main premise of (2-level) hierarchical modeling.

In our case, we wished to perform an exploratory smoothing of the PLM data that made use of as few assumptions as possible, and had easily traceable logic from underlying assumptions to the results obtained by a simple statistical inference procedure. Also, because we had adequate data at the individual level, we did not need a pooled model. These considerations meant that existing mixed-effects models were not suited to our application: they require complex inference schemes that involve approximations (because nonlinear models are generally analytically intractable) that obscure the interpretation of the results, and would be biased from the perspective of the individual [15-17].

We used a piecewise linear convex regression smoothing approach (see Multimedia Appendix 1), which can approximate smooth, nonlinear progression as a series of lines, and can also naturally model abrupt changes in progression. The only assumption about the resulting curve is that it has minimal total absolute curvature (second derivative against time) given a fixed total mean squared error with respect to the individual’s PLM data. Note that this model is related to, but much simpler than, the nonparametric spline mixed-effects model of Rice and Wu [17]. In contrast to the Rice and Wu model, however, the inference problem is convex (it has a verifiable optimum solution), which is solved by stable computations whose convergence properties are guaranteed [19].

### Residual Modeling

The trend identified above is subtracted from the UPDRS data to obtain the residuals. Modeling these fluctuations allows us to quantify the high-frequency dynamics of PD symptoms. Trends in the size of these fluctuating residuals can be detected by using a variety of methods, but because of the specifics of the trend estimation algorithm described previously, we modeled the size of the residuals against time since diagnosis by using a gamma generalized linear model (see Multimedia Appendix 1).

All analyses were carried out using specialized software written for the MATLAB platform version R2007a (MathWorks Inc, Natick, MA, USA). Creative Commons-licensed trend estimation software is distributed with this publication.

## Results

### PatientsLikeMe Dataset Validation

**Figure 1:**
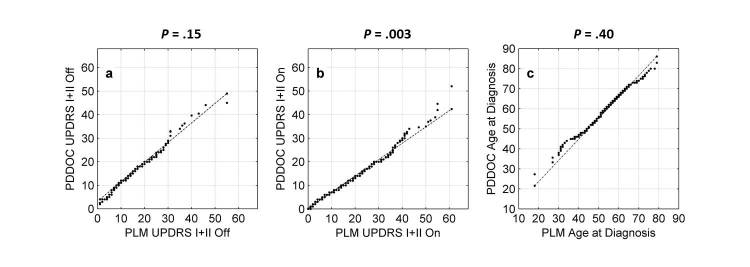
Validating the online self-reported PatientsLikeMe (PLM) dataset against the clinically scored Parkinson’s Disease Data and Organizing Center (PD-DOC) reference dataset. Visual comparisons using quantile-quantile plots; statistical comparisons using the 2-sample Kolmogorov-Smirnov (K-S) test applied to z-scored data (K-S test results displayed as the P values above graphs). (a) Unified Parkinson’s Disease Rating Scale (UPDRS) values (sum of Parts I and II) for values labeled as “off” treatment in the PLM dataset against values labeled as “off state” in the PD-DOC data; (b) as with (a), except for the “on” treatment/state labels; (c) ages at diagnosis.

The PLM and PD-DOC datasets agree in terms of the broad shape of the distribution of UPDRS values and ages at diagnosis (Figure 1). The K-S test results indicate that, up to a change in standard deviation and mean, “off” UPDRS values and ages appear to come from the same distribution, whereas “on” UPDRS values do not (Note that for the K-S test, when *P*<.05, the null hypothesis that the *z*-scored data come from the same distribution can be rejected at the 95% level). Therefore, there are some systematic differences (see discussion section), but the fact that the PLM and PD-DOC distributions are broadly similar in distribution is good evidence that the online PLM dataset is as reliable as objective clinical data about patient’s symptom severity.

### Trend Estimation

**Figure 2:**
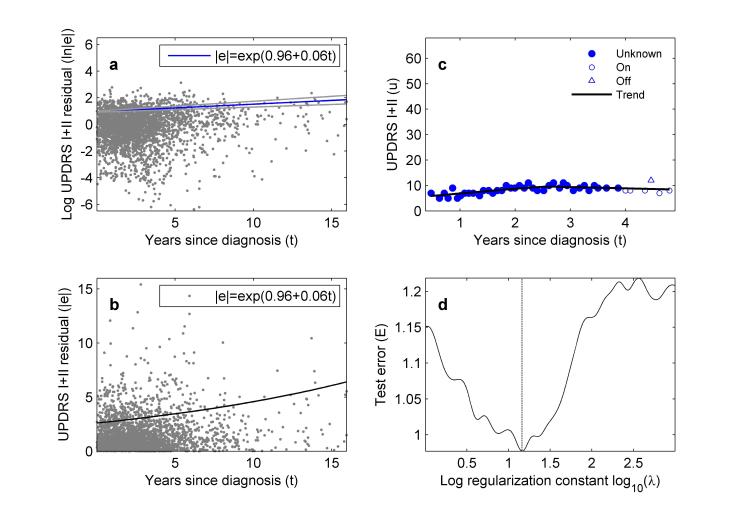
Trend fitting and residual modeling of the self-reported Unified Parkinson’s Disease Rating Scale (UPDRS) values (Parts I and II) in the PatientsLikeMe (PLM) dataset. (a) Absolute values of residuals obtained by subtracting the long- to medium-term trend from the raw values (natural logarithmic vertical scale), plotted against time since diagnosis in years. The blue line (formula inset) shows the estimated most-likely relationship between time since diagnosis and average absolute residual value. The gray lines are the 95% CI for the relationship; (b) the relationship between average absolute residual and time since diagnosis in (a) shown on a linear vertical scale; (c) UPDRS trend, used to calculate residuals, estimated from an example patient; (d) choice of trend regularization constant for (c), occurring at the smallest value of the cross-validated trend test error.

**Figure 3:**
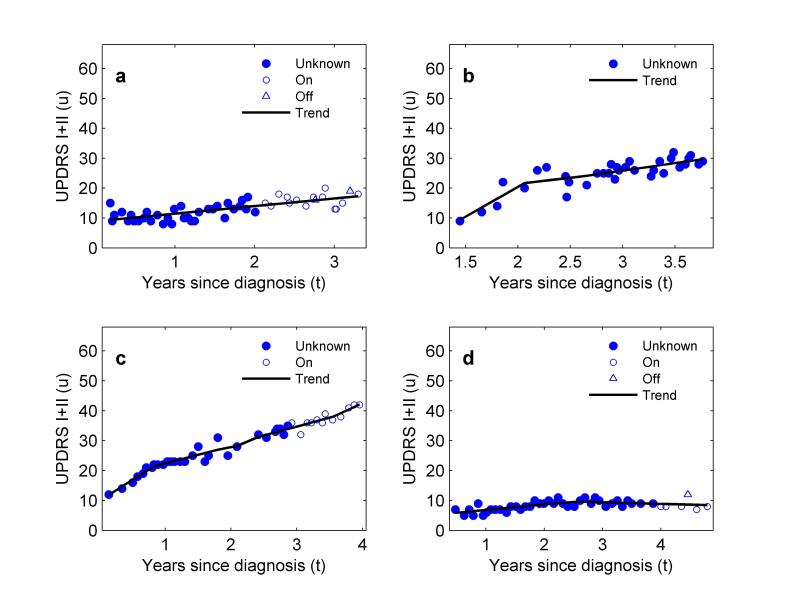
Four examples of patients in the PatientsLikeMe (PLM) dataset whose self-reported symptom dynamics conform to the current consensus picture of slow, predictable Parkinson’s disease symptom progression. Increase is generally smooth, variation around the trend (residuals) are generally small. “Unknown” refers to data in which the patient did not state whether they were on treatment (on) or off treatment (off) at the time of the symptom report.

**Figure 4:**
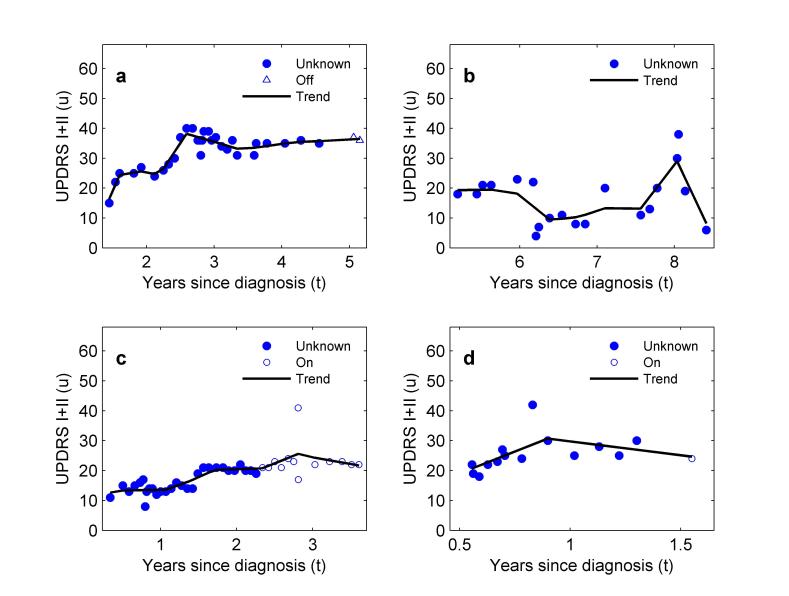
Four examples of patients in the PatientsLikeMe (PLM) dataset whose self-reported symptom dynamics diverge from the current consensus picture of smooth, gradual Parkinson’s disease progression. (a,b) Large, abrupt changes in the trend of symptom severity occurring over periods of a few weeks or months; (c,d) examples of single, large deviations from the trend in otherwise smooth progression. “Unknown” refers to data in which the patient did not state whether they were on treatment (on) or off treatment (off) at the time of the symptom report.

After performing trend estimation (Figure 2 illustrates the selection of the regularization constant and the resulting trend), our next finding is that although most patients do have smooth progression in symptom severity over time with small to moderate short-term variability (Figure 3), there are an interesting and important minority who do not (Figure 4). In the former group, we found patients with very predictable increases in symptom severity, increases that slow over time (Figure 3a, c, and d). We also see patients responding well to treatment with gradually decreasing symptom severity that eventually reaches a plateau (Figure 3b). These patients all conform to the current consensus picture of smooth, long-term symptom changes (eg, following the smooth Gompertz curve [3]). However, in the nonconforming group, we found evidence for unpredictable medium-term changes (Figure 4a and b), and occasional rapid increases (outliers) in otherwise smooth progression (Figure 4c and d).

### Residual Modeling

Residuals quantifying short-term fluctuations in symptom severity on the scale of days to weeks (which affect all patients to a lessor or greater extent) increase steadily in amplitude with time since diagnosis (Figure 2a and b). At diagnosis, average symptom severity variation is 2.6 points, rising to 5.9 points 16 years later. This finding is not simply a systematic consequence of the nonnegative UPDRS scale: if the short-term variation is to be symmetric about the long-term trend, then as the score becomes small, the residuals must get smaller to avoid negative UPDRS values. However, we find that the residuals are significantly positively skewed (*t* test against zero skewness rejected with *P*<.001, based on 1000 replicate bootstrapped skewness values). A similar argument would hold for very large UPDRS values because the scale has a maximum value of 68. The PLM data does not contain sufficient information about severely disabled patients with very high UPDRS Parts I and II values (less than 10% of the symptom reports in the database have stage 4 or 5 HY), and so this argument cannot be tested with the data available to this study.

## Discussion

### Summary of Results

This study addressed the topic of quantifying trends and variability in PD symptoms that occurs on a timescale shorter than 3 months. A dataset of 100 PD patients with symptoms, self-reported on a standard clinical scale, was analyzed. Although we have found examples of specialized studies collecting weekly UPDRS values in the literature (eg, Goetz et al [20] recording motor UPDRS weekly for 8 weeks from 16 patients to assess the effect of switching dopamine agonists), to our knowledge, this rapid self-reporting of PD symptoms stretching over many years in the PLM data is unprecedented among existing reference clinical datasets. With appropriate feedback and social network community engagement, this dataset has the potential to grow quickly at little marginal cost per patient because the usefulness of the network grows with the square of the network size (an observation known as Metcalfe’s law).

Validation demonstrated the high-frequency self-reported data are consistent with a low-frequency clinical dataset in common use in clinical PD studies. The distributions of PLM to PD-DOC off scores are essentially the same (Figure 1a). One systematic difference is that the mean age of PLM patients is approximately 6 years younger than the mean age of PD-DOC patients (Figure 1c) which is most likely a sociological effect: younger patients are generally more technologically aware, able, or willing to share their personal data. Similar patterns have been identified in other conditions, such as multiple sclerosis [21], in which the average online patient was 4 years younger than patients in a comparable clinical reference dataset.

Another systematic difference is that the symptom scores for PLM patients labeled as on treatment are biased upwards by comparison to the PD-DOC data (Figure 1b). Furthermore, the largest symptom scores (>40 UPDRS points) for PLM patients are much more common than in the PD-DOC dataset (this is the reason why, even after *z*-scoring, the K-S test fails). The most plausible explanation for this difference in on scores (PD-DOC) versus on treatment (PLM) is because of differences in interpretation of the meaning of “on.”

As discussed earlier, and repeated here for clarity, during the day there will be “on” periods when the symptoms of PD are suppressed, to a greater or lesser degree, by the treatment, and “off” periods when the full symptoms reoccur, even while taking treatment. The on/off terminology, therefore, has this somewhat specialized clinical meaning. In the PLM dataset, when completing UPDRS self-reports, patients are presented with the following question: “When you answer these questions, are you thinking about how you are on treatment or off treatment?” and they can respond by selecting either “on treatment” or “off treatment.” In the PD-DOC dataset, which is collected by trained clinical staff, it can be assumed that the on/off terminology is used according to the clinically accepted definition described previously. By contrast, with self-reporting in the PLM dataset, it is more likely that the on/off labels refer to taking treatment (on) versus not taking treatment (off), and it is unclear whether patients are generally aware of the accepted clinical meaning of the on/off terms. PLM self-reporters can indicate that this is their UPDRS value while on treatment, and this would partly concur with the clinical on state. Similarly, PLM self-reports indicating off treatment might, only partly, overlap with the clinical off state in the PD-DOC dataset.

It is likely that the PLM on label includes many scores that would be considered, clinically, as off instead, because they refer to unsuppressed symptoms occurring while the patient is actively taking treatments (the clinical off condition). This would lead to the increased scores we observed.

To identify trends in symptom progression, a cross-validated, convex piecewise linear smoothing technique was applied to the self-reported data. After subtracting the trend from the self-reported scores, the remaining residual variations appeared to increase with time since diagnosis. Furthermore, a minority of patients were shown to deviate quite considerably from the existing consensus understanding that proposes smooth, gradual change in symptoms over time. Our conclusion is that these residuals are naturally heteroscedastic.

The variations in symptom severity we detected are unlikely to be clinically irrelevant fluctuations; previous studies have estimated the minimal clinically important difference (CID) in total UPDRS (Parts I-III) values as approximately 4.1 to 4.5 points [22]. The maximum value of measuring only Parts I and II is 68, whereas the total UPDRS value is 176 points. From this, we can get a rough estimate of 1.7 (calculated as 68/176×4.3) as the minimal CID for the data in this study, which implies that at the time of diagnosis, the average residual variation of 2.6 around the trend that we found is larger than the minimal variation in symptoms needed to trigger clinical decisions. Later, at 16 years after diagnosis, the same calculation shows a moderate CID of 3.3 points, so the average variation we find here (5.9 points) could be very misleading if taken out of context (eg, in a clinical trial for a new drug treatment).

### Limitations

This study collected self-reported data about cognitive, behavioral, mood symptoms, and impairment in activities of daily living. PD is primarily a movement disorder; therefore, it is important to also be able to quantify movement symptoms. Nonetheless, activities of daily living are significantly impaired by motor deterioration, so this section of the UPDRS measures motor symptoms indirectly. Because the UPDRS is additive, even if motor symptoms increase smoothly in severity according to a long-term trend, the total UPDRS score (Parts I-III) would still show the effect of variability in Parts I and II that we observe here. Previous low-frequency studies showed evidence of the kind of variability that we found here in motor symptoms, such as bradykinesia, rigidity, and tremor [3]. Thus, we have some confidence that the explicit inclusion of a direct quantification of motor symptoms, although an important addition that would alter our assessment of the specific numerical results presented here, will not fundamentally alter our conclusions.

The PLM website has no mechanism to require patients to return to the site and enter new symptom reports, but it is possible that many patients only return to the site to enter their symptoms when they have experienced a symptom fluctuation. However, patients are unlikely to agree on what level of change in UPDRS constitutes a reportable fluctuation; and so, we would expect to see fluctuations of all sizes and differing reporting intervals in the dataset. Also, we would not expect to find regular time intervals between reports (if symptom fluctuations are indeed random). Therefore, there is no reason to believe that such fluctuation-triggered reporting is a significant source of bias in our results.

The standard CID calculations in UPDRS are performed on cross-sectional data, and refer to the symptom variation around the average across all individuals in the PD population [22]. Therefore, to draw meaningful comparisons against this literature we have performed the equivalent pooling across all individuals. These CID calculations make the statistical assumption that the patients all come from a homogenous group sharing the same UPDRS distribution. Our findings here probably indicate that this assumption may not be statistically accurate because we have found quite significant differences in symptom progression and magnitude of variation. Further statistical analysis may be needed to identify the nature of any systematic differences or subgroups in residual distribution.

### Implications for Parkinson’s Disease Research and Clinical Practice

We detected fundamental variability in symptoms on timescales less than 3 months that all patients at all stages of the disease seem to show. We note that the variability captured by the residuals we see here is not the same as the variability usually associated with fluctuators, the clinical term used to refer to patients with severe symptoms, usually in the later stages of the disease, who experience intermittent responsiveness to drug treatments [23].

Typical of many eHealth studies [24], we found a large attrition rate. Of the more than 6000 PD patients registered on the site, the fraction of sufficiently committed users is small (less than 2%). It can be estimated that entering 30 symptom reports would require, on average, approximately 7.5 hours of patients’ time in total, using timing information derived from self-administered paper data entry [5]. This is a lot of time to dedicate to entering data into a computer if there is no obvious reward (eg, financial compensation frequently used with clinical trials), even if spread over nearly 3 years, and is one plausible explanation for this severe attrition rate. It is possible that patients who are this dedicated are a select group who may introduce some, as yet unknown, bias into the results. Nonetheless, aside from this group being younger than typical clinical populations, we are not aware of any particular reason why the results we present here would be biased by focusing on a core of more dedicated symptom diarists.

We found that altering the inclusion/exclusion criteria from the PLM dataset did not lead to significant changes in the residual model.

The fundamental variability we detected here represents a critical factor in clinical decision making: knowing what sort of variability to expect is important because it determines how long to wait to detect a significant improvement in symptoms following a change in treatment regime, for example. The explicit information provided here could also be used to build improved progression models, for example, knowledge of the distribution of the residuals can be used to derive more accurate statistical model-fitting algorithms.

It is difficult to speculate on the origins of such heterogeneity in progression, but other studies have identified different clinical subtypes of PD [25]. It is possible that this might also be reflected in different progression profiles. Future research using this kind of high-frequency data might be able to identify different progression subtypes.

The main issue we identified with existing clinical PD symptom data is that it is an undersampling of the high-frequency data we presented here [26]; that is, because the sampling frequency is so low, it does not adequately represent the kind of symptom fluctuations that most patients experience on timescales shorter than 3 months.

The existence of such nonconforming patients is of critical importance to trial design in which it is typically assumed, based on current understanding of PD symptom progression, that symptoms will change slowly over the duration of the trial. However, this is not always true (Figure 4a and b). Recruiting patients into trials with the expectation that symptoms will change slowly over that period may lead to questionable results, including the failure of trial statistics to show clinically significant outcomes, not as a consequence of the failure of the treatment under test, but because of a failure to incorporate such nonconforming progression into the statistical procedures used to analyze the data.

We see this study as a prelude to the next logical step of increasing the frequency of objective symptom measurement even further. For example, we envisage these results being of utility in the design of novel, noninvasive, objective symptom severity quantification algorithms. Methods based on voice [27] or accelerometry [28], particularly using smartphones, seem promising because they offer the potential to track the effectiveness of choices in drug dosage and timing in real time. These new methods will require high-frequency reference symptom data for verification and current clinical reference data, such as the PD-DOC database, are insufficiently detailed for this purpose.

The ability to remotely self-administer tests for PD symptom severity data offers considerable cost reductions for most clinical applications by reducing the cost of clinical staff time and transport for patients during routine checkups, and lowering the costs of recruitment and tracking of patients in clinical trials, for example. Finally, there is the potential to use this kind of high-frequency data to fit models that can be used for prognostics to predict each patient’s future symptom severity.

## Multimedia Appendix 1

Trend estimation and residual modelling.

## Abbreviations

CID: clinically important difference
HY: Hoehn and Yahr
K-S: Kolmogorov-Smirnov test
PD: Parkinson’s disease
PD-DOC: Parkinson’s Disease Data and Organizing Center
PLM: PatientsLikeMe
q-q: quantile-quantile
UPDRS: Unified Parkinson’s Disease Ratings Scale
