# Supplementary material for: Quantifying Short-Term Dynamics of Parkinson’s Disease Using Self-Reported Symptom Data From an Internet Social Network
Source: J Med Internet Res. 2013 Jan 24;15(1):e20. doi: 10.2196/jmir.2112 (PMC3636067; doi:10.2196/jmir.2112)
Supplement: Supplementary file 1 [file jmir_v15i1e20_app1.pdf]

Trend Estimation by Piecewise Linear Regression Smoothing

In this section we describe the regression smoothing method used to detect the medium- and long-term trends in the self-reported UPDRS data. The method used is an adaptation of L_1 trend filtering [1], a technique that has shown to be useful in a range of smoothing problems. The following notation is used: the time since diagnosis for each patient is t_n, $n = 1,2 \dots N$, where N is the number of observations for each patient, and the combined total Part I and Part II UPDRS values is $u(t_n)$. The regression smoothing is achieved by minimizing the following functional with respect to the estimated trend $v(t_n)$:

$$E = \frac{1}{2}\|u - v\|_2^2 + \lambda\|\mathrm{D}^2 v\|_1 \qquad (1)$$

Here, the notation $\|.\|_q$ is the L_q-norm. The parameter λ is the regularization constant. For each value of λ, the output can be shown to consist of a series of straight lines joined together at their ends, ie it is a piecewise linear spline [1]. When $\lambda = 0$, the first term in the Eq. (1) dominates, so the output $v(t_n)$ is the same as the input $u(t_n)$. As λ increases, the output $v(t_n)$ becomes progressively smoother. It can be shown that there is a maximum useful value of the regularization constant $\lambda_{\max}$: if the regularization constant is equal to or larger than this, the output consists of a single, least squares straight line fit going through the data $u(t_n)$.

The matrix D^2 is a second derivative matrix that takes into account the nonuniform time spacing of the UPDRS data points. It is a tridiagonal matrix encoding a second-order accurate finite difference approximation [2]:

$$\frac{\mathrm{d}^2 v}{\mathrm{d}t^2}(t_n) \approx 2\left[\left(h_n(h_n + h_{n+1})\right)^{-1} v(t_{n-1}) - (h_n h_{n+1})^{-1} v(t_n) + \left(h_{n+1}(h_n + h_{n+1})\right)^{-1} v(t_{n+1})\right]$$

(2)

where $h_n = t_n - t_{n-1}$ is the local temporal difference. After minimization of Eq. (1) to obtain $v(t_n)$, the error residual $e(t_n) = u(t_n) - v(t_n)$ is further analyzed. Because Eq. (1) is in the form of a quadratic program, it is a convex optimization problem for which a unique, globally optimal solution is guaranteed to exist. Special optimization algorithms have been developed for such functionals, here, we use an efficient version of the primal-dual interior-point algorithm [1].

The regularization constant λ determines the smoothness of the output $v(t_n)$, and so must be chosen appropriately. In this study we use cross-validation to choose this parameter [3]. This involves a uniformly random partition of the data for each patient into a training set (80% of the data) and a testing set (the remaining 20%). Eq. (1) is optimized on the training set, then the mean absolute test error is calculated:

$$E(\lambda) = \left(\frac{1}{|Q|}\right) \sum_{m \in Q} |u(t_m) - \hat{v}(t_m)| \quad (3)$$

where Q is the set of indexes, and $|Q|$ is the number of data points, in the test partition. The test set values $\hat{v}(t_m)$ are obtained by linear interpolation/extrapolation from the smooth output points $v(t_n)$ closest in time to the test time t_m; this interpolation is justified by the piecewise linear nature of the smoothing operation. The optimal λ is the value that minimizes the test error $E(\lambda)$ (note that this is generally unique to each patient). In order to find this optimal value, we sweep across a wide range of values of λ and calculate the curve $E(\lambda)$. In order to reduce the effects of random partition sampling variation in this curve, we smooth the curve using kernel regression with Gaussian kernel of bandwidth set to 100. This makes it straightforward to find the optimal degree of smoothing for each patient. In this study, we sample 2500 values of the regularization constant over the range [0, 1000].

Gamma Generalized Linear Modeling of Residuals

In modeling the residuals of the piecewise linear regression smoothing described in section A.1 above, it is important to take into consideration the distribution of these residuals. It can be shown that minimizing Eq. (1) leads to residuals that are increasingly Laplacian, that is, they become Laplace distributed as λ increases [4,5]. From this, it follows that the absolute residuals $|e(t_n)| = |u(t_n) - v(t_n)|$ are approximately exponentially distributed (because the Laplace distribution is a symmetric, two-sided exponential). We are interested in modeling systematic variations of the absolute residuals with respect to the time since diagnosis. The sufficient statistic for the exponential distribution is the mean. Therefore, regressing the mean of the absolute residuals on the time since diagnosis allows us to make predictions about the change in distribution of the residuals over the lifetime of the patient's illness.

Least-squares linear regression is the simplest approach, but this method assumes that the residuals are Gaussian distributed, which contradicts what we know about the residuals. However, we can perform linear regression using generalized linear modeling (GLM), which allows the residuals to come from the more general class of exponential family distributions [6]. This class includes the exponential and gamma distributions as special cases. It also allows a monotonic nonlinearity (known as the link function) as part of the regression. In this study, we use the natural logarithm link function because this is the canonical choice for the gamma distribution which includes the exponential distribution as a special case:

$$\ln \mu = b_0 + b_1 t \tag{4}$$

where μ refers to the mean of the absolute residuals $|e(t_n)|$. Finding the values for the regression coefficients b_0, b_1 that maximize the likelihood of the data given the coefficients, is a convex optimization problem solvable by iteratively reweighted least squares [6].

References

1. Kim, SJ, Koh, K, Boyd, S, Gorinevsky, D. L1 trend filtering. SIAM Review 2009;51(2):339-360. DOI:10.1137/070690274

2. Sundqvist, H, Veronis, G. A simple finite-difference grid with non-constant intervals. Tellus 1970;22(1):26-31. DOI:10.1111/j.2153-3490.1970.tb01933.x

3. Hastie, T, Tibshirani, R, Friedman, JH. The elements of statistical learning: data mining, inference, and prediction. Springer Series in Statistics. New York: Springer; 2001. Isbn:0387952845

4. Boyd, SP, Vandenberghe, L. Convex optimization. Cambridge, UK: Cambridge University Press; 2004. Isbn:0521833787

5. Kubin, G. On the nonlinearity of linear prediction. IXth European Signal Processing Conference EUSIPCO’98. Rhodes, Greece; 1998.

6. McCullagh, P, Nelder, JA. Generalized linear models. Monographs on statistics and applied probability ; 37. London ; New York: Chapman and Hall; 1989. Isbn:0412317605
